# Genome-Wide Identification of the XTH Gene Family in *Carya illinoinensis* and Heterologous Expression Analysis of *CiXTH18* Under Osmotic Stress

**DOI:** 10.3390/plants15152319

**Published:** 2026-07-28

**Authors:** Junpeng Wu, Yaoyang Zhang, Hancheng Zhang, Ning Bai, Yajin Ye, Kunrong He

**Affiliations:** State Key Laboratory for Development and Utilization of Forest Food Resources, Co-Innovation Center for Sustainable Forestry in Southern China, Nanjing Forestry University, Nanjing 210037, China; junpengwu@njfu.edu.cn (J.W.); 15256646159@163.com (Y.Z.); 18175450369@163.com (H.Z.); 15090694007@163.com (N.B.); yajinye@njfu.edu.cn (Y.Y.)

**Keywords:** pecan, xyloglucan endotransglucosylase/hydrolase (XTH), XTH gene family, osmotic stress, ABA signaling

## Abstract

Drought stress severely restricts the growth and yield of woody nut crops. The xyloglucan endotransglucosylase/hydrolase (XTH) gene family participates in plant developmental and stress responses, yet its functions in pecan (*Carya illinoinensis*) under water limitation remain largely uncharacterized. Here, we performed a genome-wide identification of the XTH family in pecan, identifying 34 members distributed across four phylogenetic clades. Transcriptome-based expression analysis showed that several *CiXTH* genes responded to drought stress, among which *CiXTH18* exhibited strong and sustained induction. To investigate its function, *CiXTH18* was overexpressed in *Arabidopsis thaliana*. Under PEG-induced osmotic stress, *CiXTH18*-overexpressing lines exhibited significantly prolonged primary root lengths compared to wild-type plants. Furthermore, detached leaf assays revealed that transgenic plants had lower water loss rates under dehydration conditions. In addition, DAB staining indicated reduced H_2_O_2_ accumulation in *CiXTH18*-overexpressing plants under osmotic stress. Quantitative real-time PCR analysis revealed that the drought-responsive gene *DREB2A* and the ABA biosynthesis-related gene *NCED3* were more strongly induced in transgenic lines than in wild-type plants after PEG treatment. Promoter activity assays confirmed that *CiXTH18* was responsive to ABA treatment. Collectively, our findings indicate that heterologous expression of *CiXTH18* is associated with improved performance of Arabidopsis under PEG-induced osmotic stress and support *CiXTH18* as a promising candidate for further functional investigation.

## 1. Introduction

Drought stress is one of the most severe environmental constraints on global agricultural productivity and ecosystem stability. Water deficit disrupts cellular homeostasis, impairs photosynthesis, restricts nutrient transport, and ultimately reduces crop yield and quality. To cope with drought stress, plants have evolved to have a series of coordinated physiological, biochemical, and molecular responses, including stomatal regulation, osmotic adjustment, root system remodeling, antioxidant defense, and stress-responsive transcriptional reprogramming [1,2]. Among these adaptive mechanisms, the interaction between reactive oxygen species (ROS) signaling and abscisic acid (ABA)-mediated pathways plays a central role in coordinating drought responses [3].

Drought stress disrupts cellular redox homeostasis, leading to the accumulation of ROS such as hydrogen peroxide (H_2_O_2_) and superoxide radicals (O_2_^−^), which function both as signaling molecules and as drivers of oxidative damage [4,5]. These ROS are generated predominantly via plasma membrane-localized NADPH oxidases and the mitochondrial electron transport chain, orchestrating adaptive downstream responses while simultaneously imposing risks of oxidative damage to macromolecules [6]. Additionally, a key and rapid adaptive response to water deficit is stomatal closure, which limits transpirational water loss [7,8].

The phytohormone ABA serves as a master integrator of drought signaling by upregulating ROS-scavenging enzymes and inducing stomatal closure to conserve water [4,9]. At the mechanistic level, ABA-mediated stomatal closure involves a well-characterized signaling cascade in which PYR/PYL/RCAR receptors inhibit PP2C phosphatases upon ABA binding, thereby releasing the protein kinase OST1 to modulate guard cell ion channels and reduce turgor pressure [10,11]. Within this cascade, the bZIP transcription factor ABF2 directly binds the *TPPE* promoter, regulating root elongation and stomatal aperture through ROS-mediated signaling [12]. Notably, *NCED3*-mediated ABA biosynthesis constitutes a critical positive feedback node: ABA-activated *SnRK2* kinases upregulate *NCED3* expression, further amplifying ABA production and sustaining the signaling cascade under prolonged drought [13,14]. Together, these interconnected modules—receptor perception, kinase activation, transcriptional reprogramming, and biosynthetic amplification—form a robust and self-reinforcing drought-response network.

Despite substantial advances in understanding drought signaling pathways, the molecular basis of drought adaptation in many woody species remains poorly understood. Advances in genome sequencing and functional genomics have accelerated the identification of stress-responsive genes across diverse plants [15,16,17]. In rice (*Oryza sativa*), the transcription factor ONAC023 is strongly induced by multiple stress stimuli and positively regulates drought and heat tolerance across both vegetative and reproductive developmental stages [18]. In soybean, *GmCOL1a* is transcriptionally upregulated under salt and drought conditions; transgenic *35S::GmCOL1a* plants exhibit enhanced stress tolerance, whereas mutants display the opposite phenotype [19]. These findings collectively underscore the broad conservation of stress-response gene function across species and the potential of such genes as molecular breeding targets.

Beyond individual regulatory genes, gene families with broad roles in stress adaptation have attracted considerable research attention. The xyloglucan endotransglucosylase/hydrolase (XTH) family, which belongs to glycoside hydrolase family 16 (GH16), represents a prominent example [20]. XTH proteins catalyze the cleavage and re-ligation of xyloglucan polymers within the primary cell wall, thereby modulating wall extensibility and mechanical properties in response to developmental and environmental cues. Based on phylogenetic analyses, *XTH* genes are classified into four groups: I/II, III-A, III-B, and the early diverging group [21]. Accumulating evidence links XTH activity to a broad range of abiotic stress responses. In wheat, an *XTH* gene participates in ABA-dependent drought resistance during the tillering stage [22]. In Arabidopsis, *AtXTH26* promotes root growth under cadmium stress [23], while *AtXTH11*, *AtXTH29*, and *AtXTH33* exhibit stress-dependent transcriptional responses to combined drought and heat stress [24]. In banana (*Musa* spp.), RNA-seq analysis has implicated the XTH family in cold stress responses [25]. Genome-wide characterization of the XTH family has now been reported in numerous species, including *A. thaliana* (33 members), *O. sativa* (29), *Hordeum vulgare* (24), poplar (41), *Ananas comosus* (24), and *Schima superba* (34) [26,27,28,29,30]. Pecan (*Carya illinoinensis*), an economically important woody nut crop, is highly sensitive to prolonged water deficit during vegetative growth and kernel development [31]. Increasing drought frequency and rising global temperatures have become major constraints on pecan productivity, nut quality, and geographical cultivation stability [32]. Unlike annual crops, perennial woody plants require long-term coordination between growth maintenance and stress adaptation, yet the molecular mechanisms underlying drought resistance in pecan remain poorly understood [33]. Therefore, elucidating the role of XTH family genes in pecan drought responses is of particular importance for understanding cell wall-mediated stress adaptation.

In this study, we conducted a genome-wide analysis of the *XTH* gene family in pecan and characterized the expression patterns and potential functions of candidate drought-responsive members. We further focused on *CiXTH18*, a Group I/II XTH gene, to investigate its potential involvement in drought adaptation. We hypothesize that *CiXTH18* may correlate with plant-adaptive responses to PEG-induced osmotic stress and examine whether its promoter responds to exogenous ABA and whether its overexpression is accompanied by altered expression of ABA-related genes. Our study characterized the *CiXTH* gene family and identified *CiXTH18* as a candidate gene associated with responses to PEG-induced osmotic stress, providing a preliminary experimental basis for further molecular breeding of drought-resistant woody crops.

## 2. Results

### 2.1. Identification and Physicochemical Characterization of CiXTH Members

To comprehensively identify the XTH family in *C. illinoinensis*, we performed HMMER (V. 3.0) and BLASTP (2.14.0) searches against the pecan genome database. This initial screening identified 41 putative XTH candidates. Subsequent domain verification using the SMART and NCBI CDD databases excluded sequences lacking the conserved XTH domain (PF00722) or those encoding proteins shorter than 200 amino acids. Ultimately, 34 *XTH* genes were identified and designated *CiXTH1*- *CiXTH34* (Table S1).

The physicochemical properties of the CiXTH proteins were predicted using the ExPASy ProtParam tool, https://web.expasy.org/protparam/ (accessed on 19 August 2025) (Table S1). The predicted protein lengths ranged from 241 amino acids (CiXTH9, 25.24 kDa) to 346 amino acids (CiXTH15, 39.99 kDa). The theoretical isoelectric points (pI) varied widely from 4.69 (CiXTH13) to 9.47 (CiXTH34), suggesting considerable variation in the charge properties and interaction potentials. Notably, all CiXTH proteins exhibited a negative grand average for hydropathy (GRAVY) values, indicating that they are predominantly hydrophilic proteins (Table S1).

### 2.2. Phylogenetic Analysis of XTH Members in C. illinoinensis

**Figure 1:**
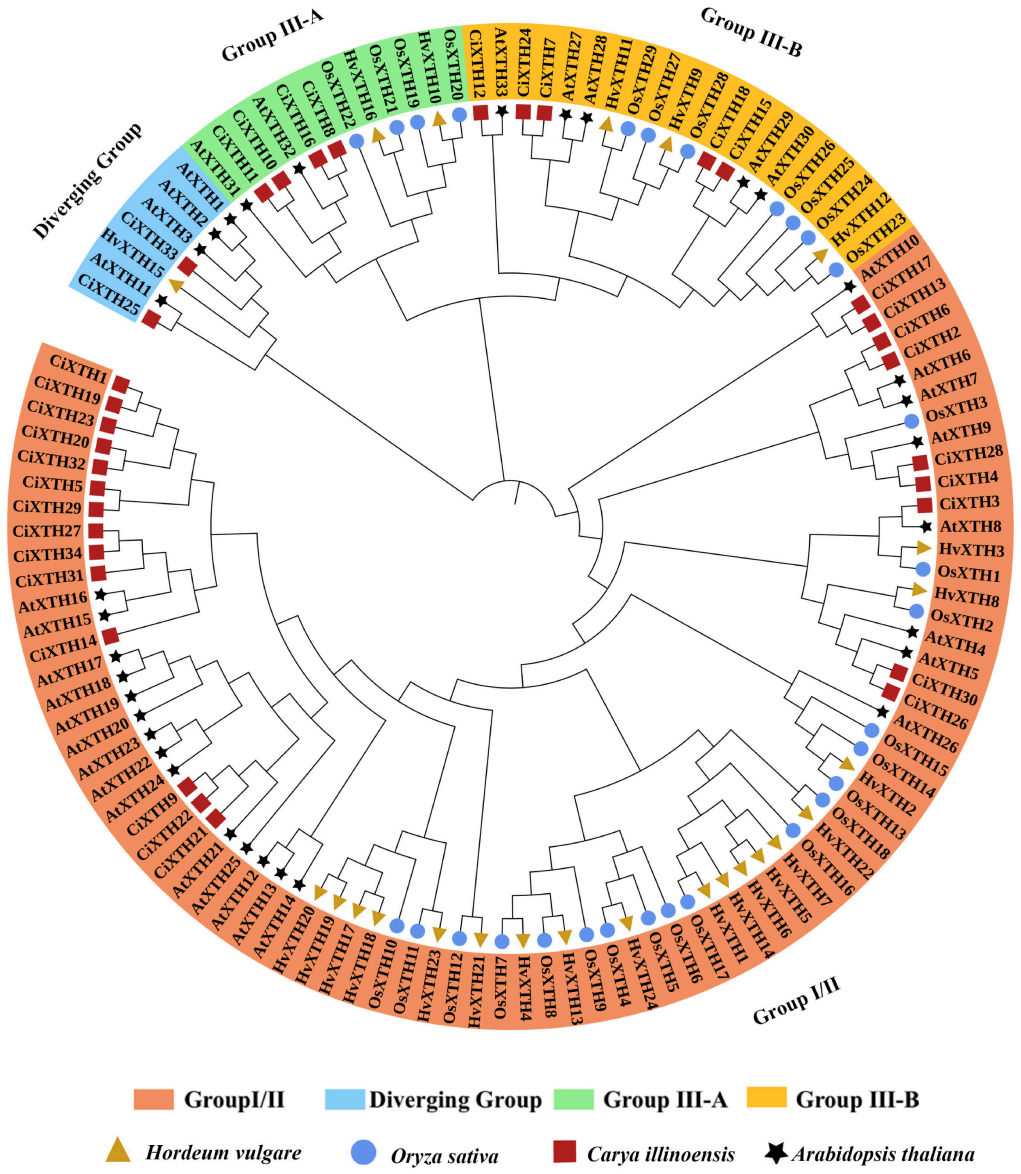
Phylogenetic classification of XTH proteins in *C. illinoinensis* and other species. The phylogenetic tree was generated based on full-length protein sequences comprising 34 members from *C. illinoinensis*, 24 from *Hordeum vulgare*, 29 from *Oryza sativa*, and 33 from *Arabidopsis thaliana*. Terminal nodes are marked with species-specific symbols: red squares (*C. illinoinensis*), yellow triangles (*H. vulgare*), blue circles (*O. sativa*), and black star (*A. thaliana*). Shaded regions delineate the four evolutionarily distinct clades: the early diverging group (blue), Group III-A (green), Group III-B (yellow), and Group I/II (orange).

To investigate the evolutionary relationships and classification of the CiXTH family, a phylogenetic tree was constructed using protein sequences from *C. illinoinensis*, *Hordeum vulgare*, *Arabidopsis thaliana*, and *Oryza sativa* (Figure 1). The resulting phylogeny classified the XTH proteins into four major clades: Group I/II, Group III-A, Group III-B, and the early diverging group. Among them, Group I/II represented the largest clade, containing 23 CiXTH members. Group III-A and Group III-B comprised four and five members, respectively. The remaining two genes, *CiXTH25* and *CiXTH33*, were assigned to the early diverging group, consistent with the evolutionary conservation of this clade within the XTH superfamily.

### 2.3. Protein Structural Features and Conserved Motifs of CiXTH Proteins

**Figure 2:**
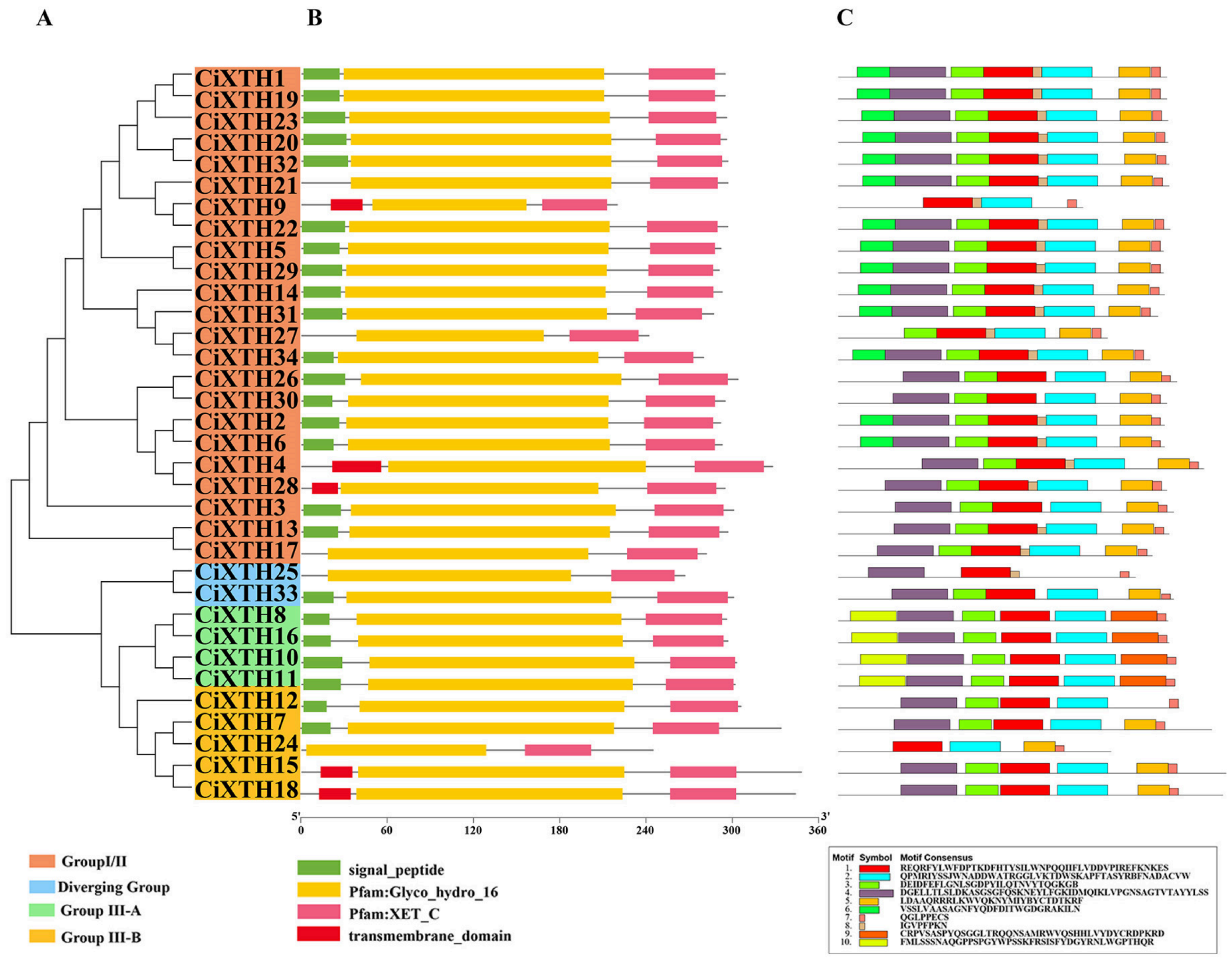
Gene structure, conserved domain architecture, and motif distribution of CiXTH family members. (**A**) The phylogenetic tree of *C. illinoinensis*; branch background colors indicate subfamily classification: orange (Group I/II), blue (Diverging Group), green (Group III-A), and yellow (Group III-B). (**B**) The conserved domain structure of each CiXTH protein, where green blocks represent signal peptides, yellow blocks represent the Glyco_hydro_16 domain, pink blocks represent the XET_C domain, and red blocks represent transmembrane domains. (**C**) The conserved motif of CiXTH proteins, with each motif depicted by a distinct color; the corresponding consensus sequences are listed in the legend.

To systematically investigate the structural organization and conserved features of CiXTH family members, conserved domain identification and motif analysis were performed. As shown in Figure 2A,B, all CiXTH proteins contained two characteristic domains: the Glyco_hydro_16 domain and the XET_C domain, which together constitute the hallmark *β*-jelly roll fold structure shared by all members of the glycoside hydrolase family 16 (GH16) [20,34]. With the exception of CiXTH4, CiXTH9, CiXTH15, CiXTH18, CiXTH21, CiXTH24, CiXTH25, CiXTH27, and CiXTH28, all other members are predicted to contain signal peptides, indicating that they may be involved in protein secretion. Notably, only CiXTH4, CiXTH9, CiXTH15, CiXTH18, and CiXTH28 were predicted to contain transmembrane domains, suggesting a potential role in membrane-associated processes. Conserved motif analysis using MEME identified ten distinct motifs across all CiXTH proteins (Figure 2C). Among these, Motif 1 and Motif 7 were universally present in all CiXTH members, suggesting their conserved function in pecan. Furthermore, the conserved catalytic domain ExDxE is absent in CiXTH9, CiXTH24 and CiXTH25, indicating that they may have undergone functional divergence during evolution.

### 2.4. Structure-Based Sequence Alignment of CiXTH Proteins

**Figure 3:**
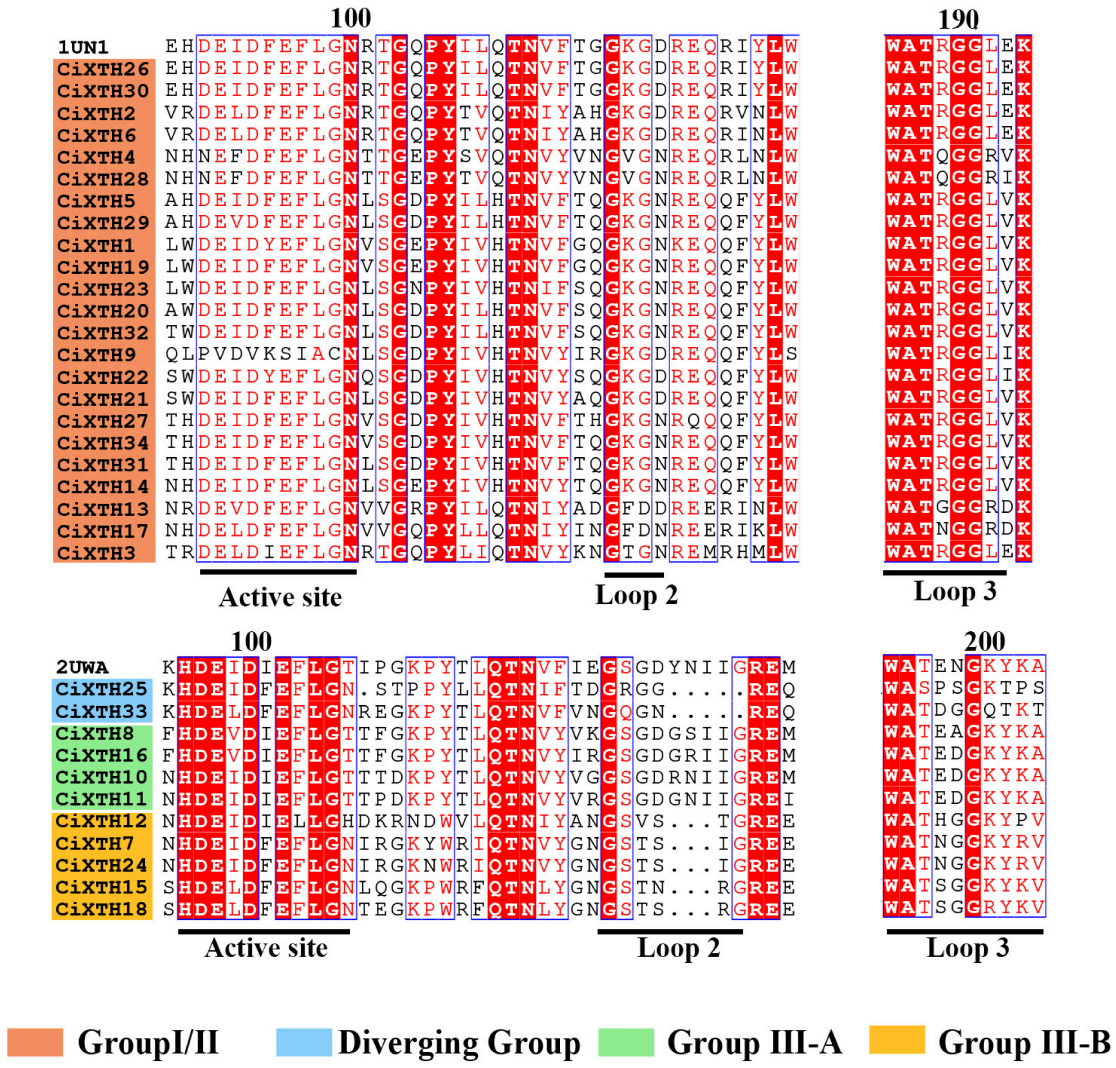
Structure-based sequence alignment of CiXTH proteins. Multiple sequence alignment of CiXTH proteins with two crystallographically resolved XTH structures, PDB ID: 1UN1 and 2UWA. Proteins highlighted with orange, blue, green, and yellow backgrounds represent Group I/II, Diverging Group, Group III-A, and Group III-B members, respectively. White letters on red boxes indicate strictly conserved residues, red letters on white boxes indicate conserved similarities, and blue frames indicate conserved regions. The conserved active site (ExDxE) and the functionally important Loop 2 and Loop 3 regions are indicated below the alignment.

To further characterize the structural features of CiXTH proteins, structure-based sequence alignment was performed, with two crystallographically resolved XTH structures serving as references: PttXET16-34 (PDB ID: 1UN1) representing Group I/II and TmNXG1 (PDB ID: 2UWA) representing Group III-A. As shown in Figure 3, all CiXTH proteins except for CiXTH9 harbored the conserved catalytic ExDxE motif, in which the first glutamic acid residue functions as a catalytic nucleophile to initiate the enzymatic reaction, while the second glutamic acid residue serves as a general acid–base catalyst that facilitates substrate binding and cleavage [29]. Structural comparison revealed that Loop 2, a region previously demonstrated to play a pivotal role in determining xyloglucan endotransglucosylase (XET) versus xyloglucan endohydrolase (XEH) enzymatic activity [35], was markedly extended in Group III-A members relative to other groups. Furthermore, the Loop 3 sequence WATRGG, which was conserved across most Group I/II members, was replaced by WATxxG in Group III-A members, suggesting subfamily-specific structural adaptation with potential functional implications.

### 2.5. Drought-Responsive Expression Profiling of CiXTH Genes

**Figure 4:**
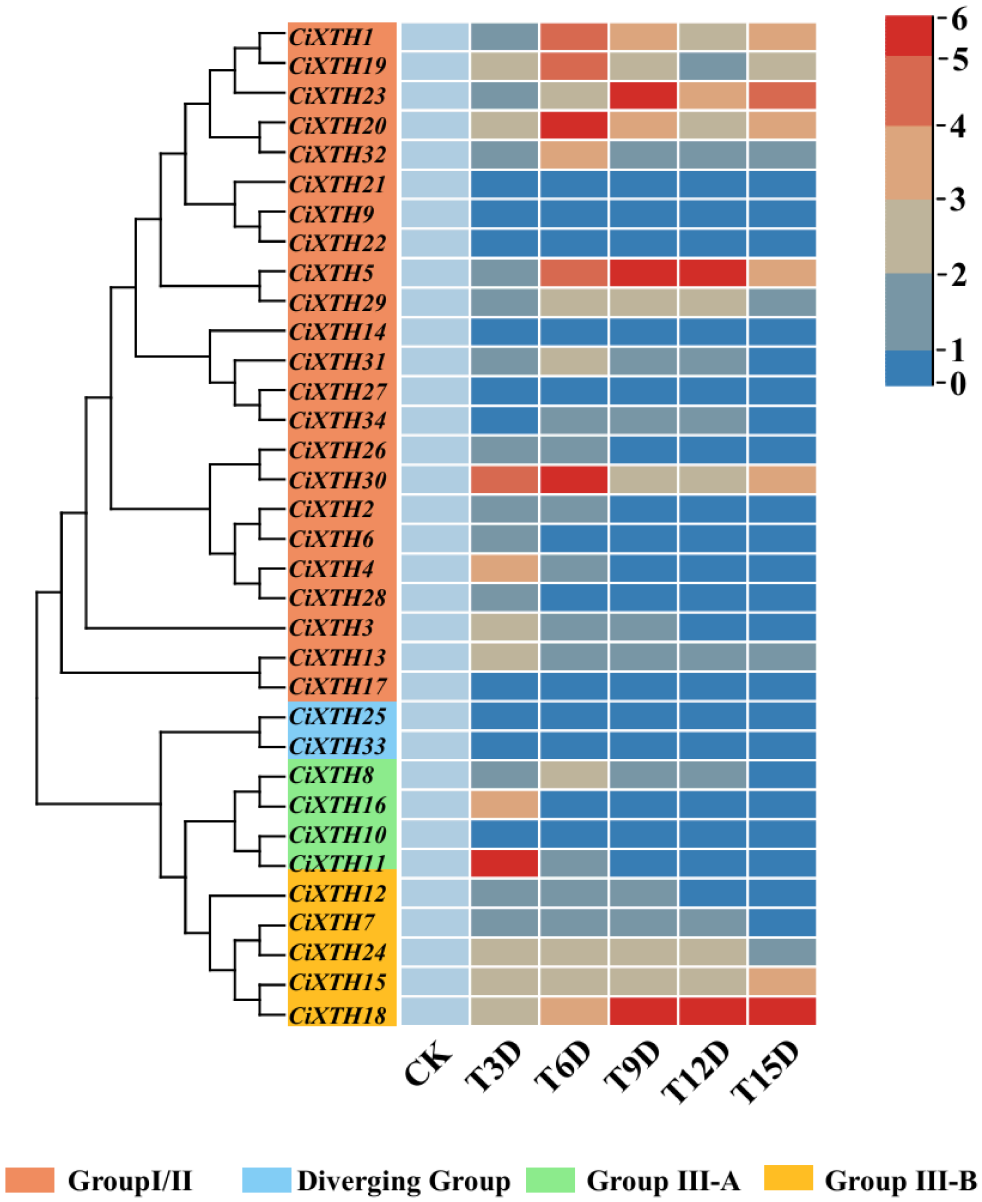
Expression profiles of *CiXTH* genes in *C. illinoinensis*. Heatmap showing the expression levels of *CiXTH* genes under drought stress at 0, 3, 6, 9, 12, 15 days. The relative expression level data of the heat map is based on the fold change in FPKM values. The colored labels on the left indicate the four evolutionary clades consistent with Figure 1: early diverging group (blue), Group III-A (green), Group III-B (yellow), and Group I/II (orange).

To prioritize candidate genes for functional characterization, we analyzed publicly available transcriptomic data from drought-treated pecan [36], visualized in a heatmap (Figure 4). Expression changes were quantified based on fold-change values. The *CiXTH* genes exhibited distinct expression patterns following drought treatment. Notably, eight genes (*CiXTH9*, *CiXTH14*, *CiXTH17*, *CiXTH21*, *CiXTH22*, *CiXTH25*, *CiXTH27*, and *CiXTH33*) showed no detectable expression across all examined time points, suggesting they may be transcriptionally inactive under these conditions or possess highly specific spatiotemporal expression patterns that are not captured in this dataset. In contrast, the remaining 26 genes displayed varying degrees of drought-responsive expression. Among them, *CiXTH18* showed a progressive increase in expression with prolonged drought exposure. This expression trend, combined with its close phylogenetic clustering with *AtXTH29*, a gene previously characterized as being involved in drought stress [24], suggests that *CiXTH18* exhibits strong drought inducibility and identifies it as a candidate for further functional analysis under drought-related stress conditions.

### 2.6. Heterologous Expression of CiXTH18 Is Associated with Improved Performance Under PEG-Induced Osmotic Stress in Arabidopsis

**Figure 5:**
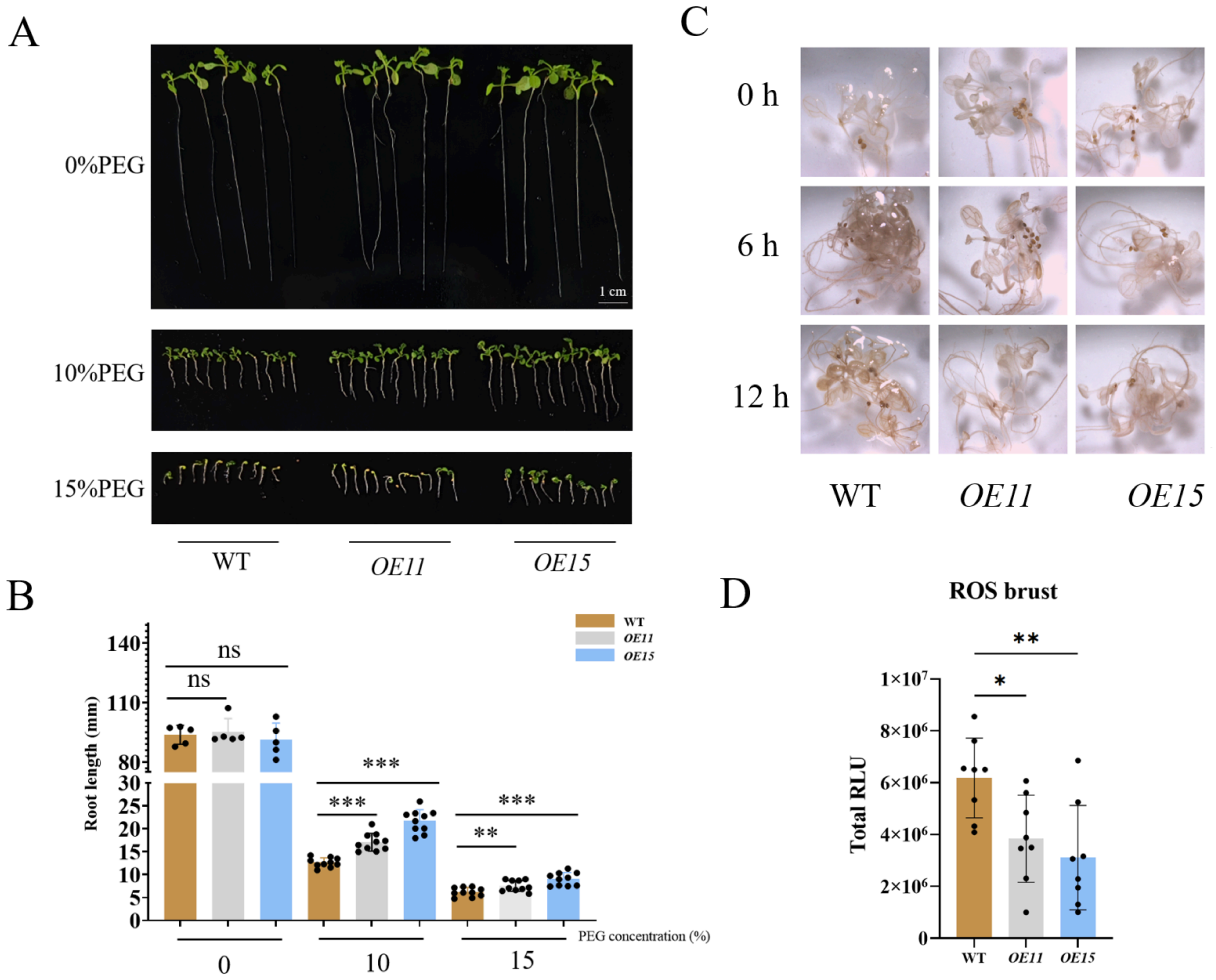
Heterologous *CiXTH18* expression correlates with longer primary roots and reduces H_2_O_2_ accumulation under osmotic stress. (**A**) Phenotypic comparison of wild-type (WT) and transgenic lines *OE11* and *OE15* grown on 1/2 MS medium supplemented with 0% (Control), 10%, and 15% PEG 6000 for 10 days. (**B**) Quantitative analysis of primary root lengths under different PEG concentrations. Root length measurements were performed on 5 seedlings under the 0% PEG osmotic stress treatments, whereas 10 individual seedlings were quantified for the 10% and 15% PEG osmotic stress treatments, respectively. (**C**) DAB staining of H_2_O_2_ accumulation in leaves of WT and transgenic lines after 15% PEG treatment for 0, 6, and 12 h. Darker brown staining indicates higher ROS levels. (**D**) ROS production in the WT, OE11 and OE15 measured as relative luminescence units (RLU) within 6 h under 15% PEG osmotic stress. As for RLU measurement, 8 individual seedlings were quantified. All data were analyzed by one-way ANOVA with Tukey’s test (* *p* < 0.05, ** *p* < 0.01, *** *p* < 0.005), ns represents no difference.

Given the strong drought inducibility of *CiXTH18*, transgenic Arabidopsis plants overexpressing *CiXTH18* under the CaMV 35S promoter were generated for functional characterization. Following the identification of T3 homozygous lines, qPCR analysis revealed that *CiXTH18* was most highly expressed in the *OE11* and *OE15* transgenic plants (Figure S1); these two lines were selected for all subsequent experiments. To assess the performance of the transgenic plants under PEG-induced osmotic stress, seeds of WT, *OE11* and *OE15* were germinated on 1/2 MS medium supplemented with 10% and 15% PEG 6000. As shown in Figure 5A and Figure 5B, under 10% PEG-induced osmotic stress, the root lengths of *OE11* and *OE15* transgenic lines were increased by 35% and 71%, respectively, compared to those of WT plants. Under more severe stress conditions (15% PEG), the root lengths of *OE11* and *OE15* remained significantly greater than those of WT, exhibiting increases of 21.5% and 45.4%, respectively (Figure 5A and Figure 5B). These results indicate that *CiXTH18* heterologous expression appears to correlate with longer primary root length under PEG-mediated osmotic stress.

To further examine oxidative responses, 7-day-old seedlings were subjected to 15% PEG treatment for 0 h, 6 h and 12 h, followed by 3,3′-diaminobenzidine (DAB) staining to detect H_2_O_2_ accumulation (Figure 5C). Under control conditions (0 h), no obvious differences in staining intensity were observed between WT and transgenic lines. However, following 6 and 12 h of osmotic stress, WT leaves exhibited stronger DAB staining compared with *OE11* and *OE15* lines, indicating excessive H_2_O_2_ accumulation. In addition, total ROS accumulation triggered by 15% PEG-induced osmotic stress was quantified via a luminol-HRP chemiluminescence assay, with continuous luminescence signals recorded over a 6 h treatment period using a microplate reader. As shown in Figure 5D, the total of relative luminescence units (RLU) was markedly higher in wild-type (WT) seedlings than in *OE11* and *OE15*. These results indicate that *CiXTH18*-overexpressing lines accumulated less ROS than WT plants under PEG-induced osmotic stress.

### 2.7. CiXTH18-Overexpressing Lines Exhibit Reduced Water Loss from Detached Leaves

**Figure 6:**
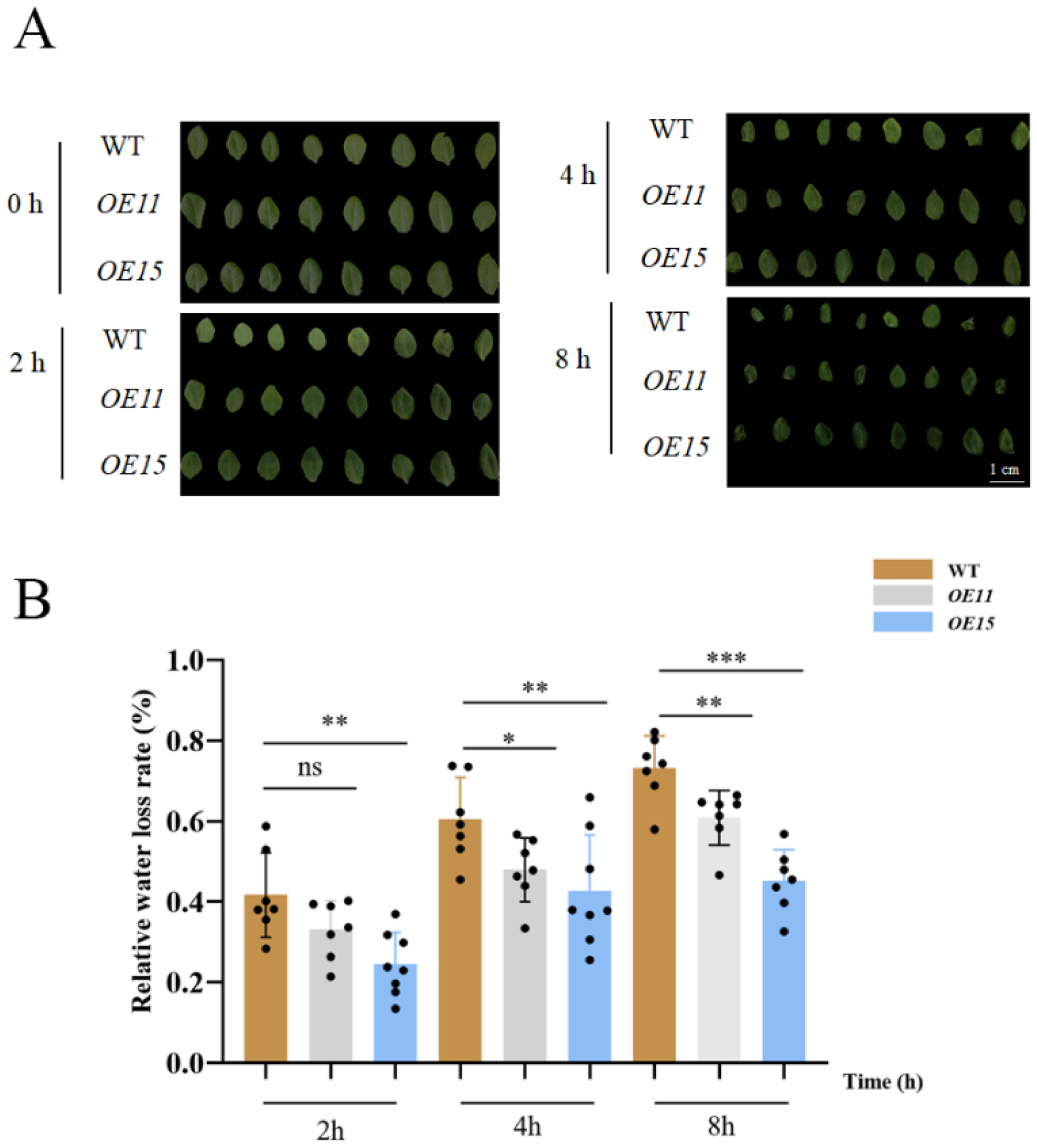
*CiXTH18*-overexpressing lines exhibit reduced water loss during detached-leaf dehydration. (**A**) Detached leaf dehydration of WT, *OE11*, and *OE15* in room temperature at 0, 2, 4, and 8h. Bar = 1 cm. (**B**) Water loss rates of detached leaves from WT, *OE11*, and *OE15* measured at 0, 2, 4, and 8 h. Data are presented as means ± SEM, *n* = 8. Asterisks indicate significant differences compared with WT. All data were analyzed by one-way ANOVA with Tukey’s test (*, *p* < 0.05, **, *p* < 0.01, ***, *p* < 0.005), ns represents no difference.

To evaluate the effect of *CiXTH18* on transpirational water loss, detached leaves from WT, *OE11*, and *OE15* plants were subjected to dehydration at room temperature, and the fresh weights were recorded at 0, 2, 4, and 8 h. As shown in Figure 6A,B, no obvious phenotypic differences were observed between WT and *OE11* at 2 h, although the *OE15* showed a slightly reduced water loss rate. At 4 and 8 h, WT leaves showed more severe wilting and higher water loss rates than *OE11* and *OE15*, whereas both transgenic lines exhibited lower dehydration-associated weight loss.

### 2.8. Expression of Drought- and ABA-Related Genes in CiXTH18-Overexpressing Lines Under PEG Treatment

**Figure 7:**
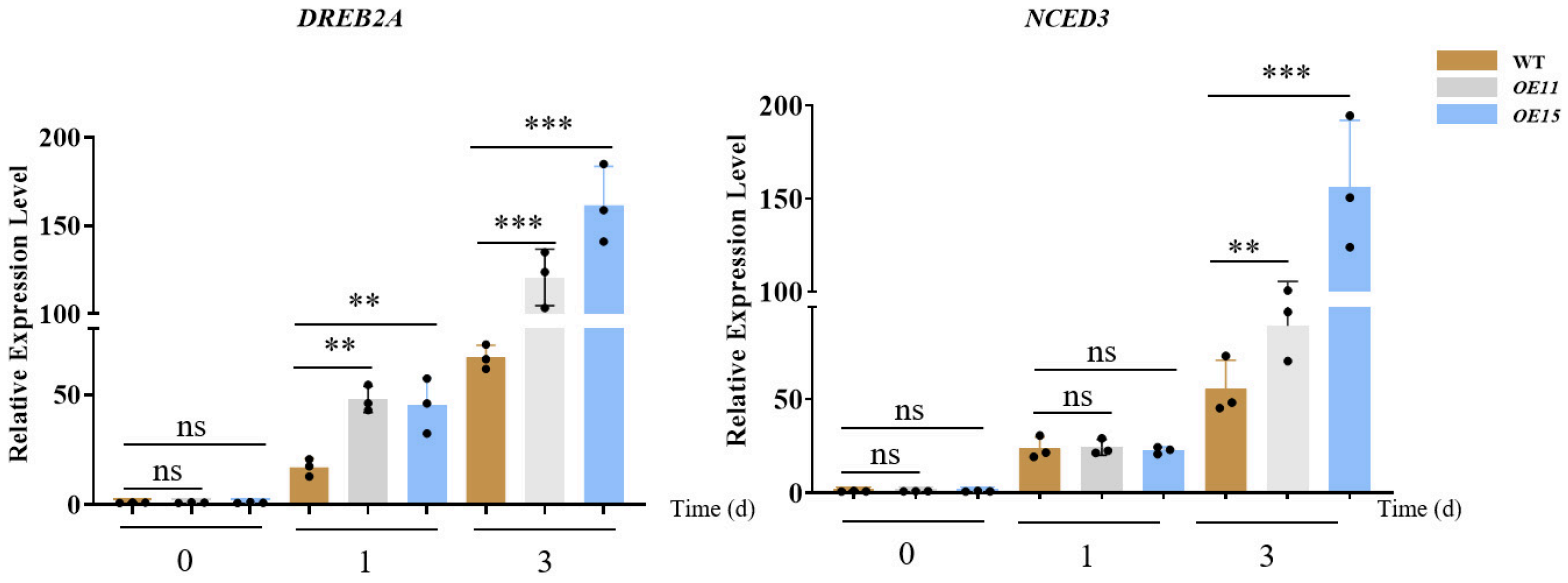
Expression analysis of drought-responsive genes in WT, *OE11*, and *OE15*. Relative expression levels of *DREB2A* and *NCED3* in WT, *OE11*, and *OE15* plants after 15% PEG treatment for 0, 1, 3 days. Gene expression levels were normalized to reference *EF1a* gene and are presented relative to WT at 0 d. Three biological samples were measured and each point in the figure represents the average of the three technical replicates. Asterisks indicate significant differences compared with WT. All data were analyzed by one-way ANOVA with Tukey’s test (**, *p* < 0.01, ***, *p* < 0.005). ns indicates no significant difference.

To examine transcriptional responses associated with *CiXTH18* overexpression under PEG-induced osmotic stress, the transcript levels of key drought-responsive genes were analyzed by qPCR in WT and transgenic plants after treatment with 15% PEG for 0, 1, and 3 days (Figure 7). *DREB2A*, an important drought-responsive transcription factor, was markedly induced by PEG treatment in WT plants. Notably, its expression levels were significantly higher in *OE11* and *OE15* than in WT after PEG treatment, indicating that *CiXTH18*-overexpressing lines exhibited a stronger transcriptional response to osmotic stress.

Similarly, the expression of *NCED3*, which encodes a key enzyme involved in ABA biosynthesis, was also induced under PEG treatment. Although no significant difference in *NCED3* expression was observed between WT and transgenic lines after 1 day of treatment, its expression levels were significantly higher in *OE11* and *OE15* than in WT after 3 days (Figure 7). These results indicate that *CiXTH18* overexpression is associated with stronger induction of *DREB2A* and *NCED3* under PEG treatment, although a direct regulatory relationship remains to be established.

### 2.9. ABA Enhances the Promoter Activity of CiXTH18

**Figure 8:**
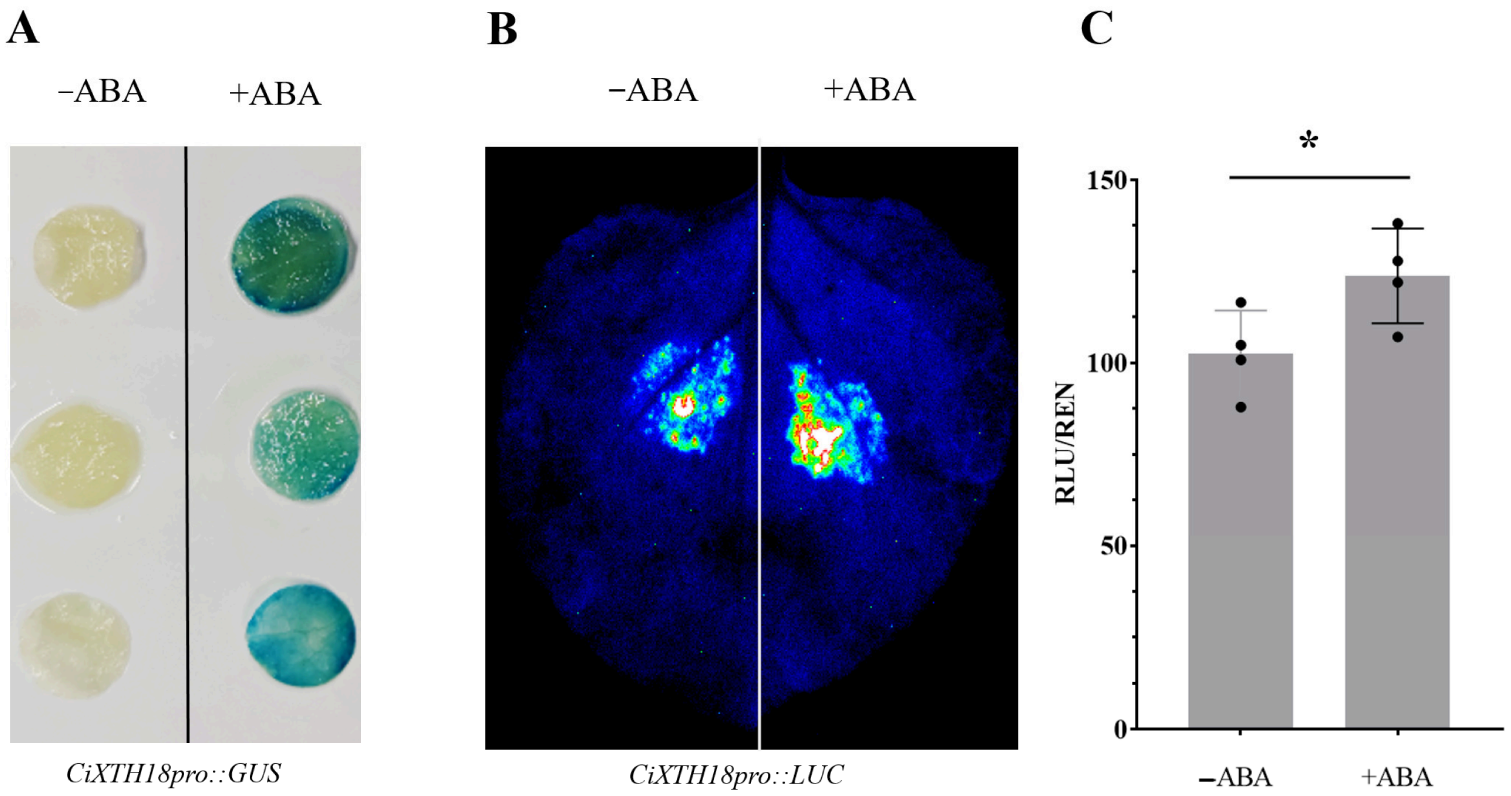
ABA-responsive activity of the *CiXTH18* promoter. (**A**) Histochemical GUS staining of tobacco leaves infiltrated with the agrobacterial strains containing *CiXTH18pro::GUS.* The bluer the color, the higher the activation level. (**B**) Representative luciferase luminescence image of tobacco leaves infiltrated with the agrobacterial strains containing *CiXTH18pro::LUC.* (**C**) The luminescence intensity in *Agrobacterium*-infiltrated tobacco leaves. ABA treatment was performed using 100 μM ABA for 24 h. Four biological samples were measured and each point in the figure represents the relative fluorescence intensity. Asterisks indicate significant differences compared with WT. All data were analyzed by *t* test (* *p* < 0.05).

Given the pivotal role of ABA in orchestrating drought stress responses, we investigated whether *CiXTH18* is transcriptionally regulated by ABA. A 2000 bp promoter fragment upstream of the *CiXTH18* start codon was cloned and fused to *β*-glucuronidase (GUS) and firefly luciferase (LUC) reporter genes, independently. The resulting constructs were transiently expressed in *Nicotiana benthamiana* leaves (Figure 8). GUS staining revealed markedly intensified blue coloration in leaves carrying the *ProCiXTH18::GUS* construct following 100 µM ABA treatment relative to mock controls. Concurrently, LUC assays showed a substantial increase in luminescence intensity upon ABA treatment in *ProCiXTH18::LUC*-expressing leaves. Together, these results indicate that the *CiXTH18* promoter responds to exogenous ABA in the transient *N. benthamiana* assay.

## 3. Discussion

Plant cell wall remodeling represents a central adaptive mechanism through which plants respond to environmental stresses. Drought, in particular, imposes profound constraints on cellular turgor, hydraulic conductivity, and the maintenance of tissue integrity. The XTH family, which governs the remodeling of xyloglucan–cellulose networks within the primary cell wall, plays a pivotal role in modulating cell wall extensibility and mechanical stability during stress adaptation [37,38,39]. In the present study, we performed a comprehensive genome-wide identification of *XTH* genes in *C. illinoinensis* and identified 34 *CiXTH* members. Phylogenetic classification grouped these genes into four clades, consistent with the evolutionary architecture of XTH families reported in other plant species [40,41]. The expansion of Group I/II members observed in pecan is particularly notable, as this clade has been widely associated with stress-responsive cell wall remodeling and rapid cell elongation processes. Similar patterns of expansion have been documented in several woody species, suggesting that diversification of *XTH* genes may represent an evolutionary strategy enabling perennial plants to adapt to fluctuating environmental conditions [39].

Transcriptomic analysis revealed that a substantial proportion of *CiXTH* genes responded to drought, underscoring the importance of this family in drought-associated regulatory networks. Among these, *CiXTH18* exhibited the most pronounced and sustained transcriptional induction, making *CiXTH18* a suitable candidate for further functional analysis under drought-related stress conditions. Consistent with this, recent studies in other species have demonstrated that *XTH* genes contribute to abiotic stress tolerance through the coordination of cell wall plasticity and physiological adaptation. For instance, overexpression of *MdXTH30* significantly enhanced drought and salt tolerance in apple by modulating stress-responsive signaling pathways [42]. Similarly, functional analyses in poplar demonstrated that *PagXTH12* contributes to drought resistance through regulating biomass allocation and stress adaptation [41]. These studies collectively support the notion that *XTH*-mediated cell wall restructuring plays a fundamental role in enabling plants to maintain growth and structural integrity under water deficit conditions.

Heterologous expression analysis showed that *CiXTH18*-overexpressing Arabidopsis lines exhibited several distinct responses under PEG-induced osmotic stress. Transgenic plants overexpressing *CiXTH18* exhibited significantly enhanced primary root elongation under PEG-induced osmotic stress. Root architecture is a critical determinant of drought adaptation, as deeper and more extensive root systems allow plants to access water from deeper soil layers [43]. The enhanced root growth observed in *CiXTH18*-overexpressing plants may therefore represent an adaptive strategy that facilitates root system development under osmotic stress. This finding aligns with previous studies demonstrating that cell wall remodeling enzymes, including XTHs and expansions, play key roles in regulating root elongation under abiotic stress conditions [43,44]. By facilitating xyloglucan restructuring within the cell wall matrix, XTH enzymes enable controlled cell wall loosening, which is essential for cell expansion and root growth under osmotic stress [45]. Therefore, the enhanced root elongation observed in *CiXTH18*-overexpressing lines may be related to altered cell wall remodeling capacity under osmotic stress. However, because XTH enzymatic activity and cell wall composition were not directly measured in this study, further biochemical and cytological analyses are needed to clarify how *CiXTH18* affects root cell expansion.

Another notable finding of this study is that *CiXTH18*-expressing Arabidopsis lines exhibited attenuated H_2_O_2_ accumulation following PEG treatment. Excess ROS triggered by osmotic stress acts as cytotoxic agents that damage cellular macromolecules [46,47,48]. Mechanistically, severe water deficit disrupts cell wall integrity and triggers apoplastic ROS overproduction, where H_2_O_2_ acts as a primary cytotoxic molecule to exacerbate membrane lipid peroxidation and accelerate cellular dehydration [49]. Excessive H_2_O_2_ further disrupts cell wall mechanical stability and facilitating passive water leakage from mesophyll cells; this cascade creates a self-amplifying vicious cycle linking oxidative burst and dehydration injury [50]. Consistent with our DAB staining and ROS chemiluminescence measurement results, overexpression of the *OsDUF2488* gene can enhance the ability of ROS decomposition, thereby improving tolerance to dehydration and oxidative stress, and can synergistically regulate redox homeostasis and promote stress tolerance in rice with *OsPrx1.1* [51]. The reduced ROS levels observed in transgenic plants suggest that *CiXTH18* may indirectly enhance antioxidant capacity or stabilize redox homeostasis under stress conditions, though the precise biochemical mechanism linking cell wall remodeling to cytoplasmic ROS regulation warrants further investigation.

In parallel, fine-tuned ROS homeostasis is also central to balancing plant growth and stress adaptation, as exemplified by the miR408–plantacyanin regulatory module, which modulates guard cell ROS pools to coordinate drought-associated physiological adjustments [48]. *CiXTH18*-overexpressing lines exhibited lower water loss during detached-leaf dehydration. Stomatal regulation is the primary physiological mechanism controlling leaf water status during drought [52]. Similar connections between cell wall modification and stomatal movement have been proposed in previous studies, as guard cell wall flexibility is essential for dynamic changes in stomatal aperture. Nevertheless, the present data do not demonstrate a direct role of *CiXTH18* in guard cell wall remodeling. Quantitative analysis of stomatal aperture, guard cell wall properties, and cell wall-related components would further strengthen this conclusion. Although detached-leaf assays demonstrated reduced water loss in *CiXTH18*-overexpressing plants, this approach cannot fully represent whole-plant water relations under drought conditions. Future studies incorporating measurements of relative water content, leaf water potential, transpiration rate, and root water uptake will be necessary to further clarify the contribution of *CiXTH18* to plant water status regulation.

Plasma membrane-localized NADPH oxidases AtrbohD and AtrbohF constitute the major enzymatic sources of ABA-triggered ROS in guard cell signaling, establishing a core functional connection between ABA perception and H_2_O_2_ generation under water deficit [53]. Downstream of H_2_O_2_ accumulation, hydrogen peroxide activates plasma membrane calcium-permeable channels, which are indispensable for full ABA-mediated stomatal signaling and stress responses [54]. In addition to the reduced leaf water loss observed in detached-leaf assays, RT-qPCR demonstrated significantly higher transcript abundance of *DREB2A* and *NCED3* in CiXTH18-overexpressing lines following PEG-induced osmotic stress. *DREB2A* is a well-documented transcription factor governing transcriptional reprogramming in response to water limitation [55,56], whereas *NCED3* encodes a rate-limiting enzyme in ABA biosynthesis and plays a crucial role in drought-induced ABA accumulation [4,57,58]. Previous studies have shown that several *XTH* genes contain ABA-responsive elements in their promoters and are transcriptionally activated by ABA signaling [59]. For example, ABA treatment induces the expression of multiple *XTH* genes in rice and wheat during drought stress, indicating that these enzymes may act downstream of ABA signaling pathways [40,60]. Combined with the ABA-inducible activity of the *CiXTH18* promoter confirmed by dual reporter assays, these transcriptional data imply that *CiXTH18* may participate in ABA-dependent signaling cascades triggered by osmotic stress. Nevertheless, direct biochemical and genetic evidence is still required to define the precise position of *CiXTH18* within ABA signaling pathways and to establish whether it acts upstream or downstream of ABA biosynthesis.

Collectively, our results outline a potential multi-layered association between *CiXTH18*, ROS homeostasis and ABA signaling under PEG-mediated osmotic stress. The present data do not yet establish *CiXTH18* as a central regulatory factor of stress responses, and further mechanistic work is required to validate its direct biological functions. Concurrently, *CiXTH18* reduces ROS accumulation and decreases dehydration-associated water loss, contributing to improved cellular protection and water retention. These physiological changes are accompanied by enhanced activation of key drought-responsive genes, including *DREB2A* and *NCED3*, suggesting that *CiXTH18* may function at the interface between cell wall dynamics and stress signaling pathways. Such integration of structural and signaling processes is increasingly recognized as a hallmark of plant stress adaptation. Given the increasing frequency of drought events associated with climate change, the identification of *CiXTH18* as a promising candidate of osmotic stress provides valuable insights into the molecular mechanisms underlying stress adaptation in pecan and highlights this gene as a promising candidate for molecular breeding programs aimed at improving drought resilience in woody plants.

## 4. Materials and Methods

### 4.1. Plant Materials and Root Length Measurement

Pecan samples were collected from Jurong, Jiangsu Province, China. *A. thaliana* and tobacco (*Nicotiana benthamiana*) seeds were surface-sterilized in a solution containing 10% (*w*/*v*) bleach and 0.05% (*w*/*v*) Tween 20 (Nanjing, China) for 15 min, followed by five rinses with double-distilled water. The sterilized seeds were stratified at 4 °C for two days and then transferred to a growth chamber (Nanjing, China) under a 16 h light/8 h dark photoperiod at 23 °C. Four-week-old *Arabidopsis* and tobacco were used for transformation and infiltration experiments, respectively.

For in vitro osmotic stress assays and root length measurement, sterilized *Arabidopsis* seeds were sown on 1/2 Murashige and Skoog (MS) (Nanjing, China) medium [61] supplemented with 10% and 15% (*w*/*v*) PEG 6000 (Nanjing, China). Phenotypic characteristics were assessed and quantified at 10 days post-germination. After photographing the seedlings, imageJ software, https://imagej.net/ij/download.html (accessed on 23 August 2025) was used to measure root lengths. Five seedlings were analyzed for the 0% PEG control group, and ten individual seedlings were quantified for each of the 10% and 15% PEG treatments. For soil-based drought stress experiments, four-week-old seedlings maintained under full-watered conditions were subjected to stress via irrigation with a 15% PEG solution. Samples were harvested after 1 d and 3 d treatment and stored at −80 °C for subsequent qPCR (Nanjing, China) analysis.

### 4.2. Identification and Characterization of XTH Family Members in C. Illinoinensis

The genome sequence of *C. illinoinensis* was retrieved from the NCBI database, https://www.ncbi.nlm.nih.gov/ (accessed on 19 August 2025). Hidden Markov Model (HMM, V. 3.0) profiles corresponding to XTH family domains (PF00722 and PF06955) were obtained from the Pfam database, http://pfam.xfam.org/ (accessed on 19 August 2025). HMMER software was employed to scan the *C. illinoinensis* protein sequences for these domains, using a significance threshold of 1 × 10^−5^ [62]. Candidate XTH proteins were further verified using the SMART database, https://smart.embl.de/ (accessed on 19 August 2025) [63], and redundant sequences were removed manually. The physicochemical properties of the identified CiXTH proteins, including molecular weight (MW), isoelectric point (pI), and protein length, were predicted using the ExPASy ProtParam tool [64].

### 4.3. Phylogenetic Analysis of CiXTH Genes

*A. thaliana* proteins were downloaded from the TAIR website https://www.A.thaliana.org/ (accessed on 19 August 2025). Proteins of *H. vulgare* and *O. sativa* were downloaded from the NCBI website https://www.ncbi.nlm.nih.gov/ (accessed on 19 August 2025). These sequences were aligned with ClustalW 2.1 software. The phylogenetic tree was constructed using MEGA 11 software using the neighbor-joining method with the following parameters: Poisson model, pairwise deletion, and 1000 bootstrap replicates [65]. The iTOL website (https://itol.embl.de/upload.cgi) was used to modify the phylogenetic tree, accessed on 20 May 2025.

### 4.4. Expression Patterns Analysis of CiXTH Genes

Expression levels of *CiXTH* genes were investigated by re-analyzing publicly available transcriptomic data from drought-treated pecan [36]. The FPKM values of *CiXTH* genes were extracted from the transcriptome dataset and used to evaluate their transcriptional responses under drought stress. To visualize relative expression changes, fold-change values were calculated based on the expression levels of drought-treated samples relative to the corresponding control samples. A heatmap was generated using TBtools v2.303 to display the expression patterns of *CiXTH* genes under drought treatment.

### 4.5. RNA Extraction and qRT-PCR Analysis

Total RNA was isolated from plant tissues using TRIzol reagent according to the manufacturer’s instructions, and 500 ng of total RNA was used as template to synthesize the first-strand cDNA by reverse transcription, using the PerfectStart Uni RT&qPCR Kit (Thermo Fisher Scientific, Shanghai, China). qPCR was performed using the QuantStudio One (Thermo Fisher Scientific, Shanghai, China) with the PerfectStart Uni RT&qPCR Kit.

For RT-qPCR analysis, 4-week-old WT, *OE11* and *OE15* Arabidopsis plants were treated with 15% PEG for 0, 1, and 3 days, respectively. Leaves were collected at each time point and immediately frozen in liquid nitrogen. Three independent biological replicates were prepared for all sampling groups. *ELF1a* was used as the reference gene. Relative expression levels were calculated using the 2^−ΔΔCT^ method. The primers used for RT-qPCR analysis are listed in Table S1.

### 4.6. Arabidopsis Transformation and ROS Accumulation

The coding sequence of *CiXTH18* was isolated from the *C. illinoinensis* genome, yielding a 1029 bp open reading frame (ORF). This gene was cloned into a modified pCAMBIA1305 vector with a GFP tag. The recombinant vector was introduced into the *Agrobacterium tumefaciens* GV3101 strain for *Arabidopsis* transformation.

To detect ROS accumulation, 3,3′-diaminobenzidine (DAB) staining was performed according to previously described methods [66]. Seven-day-old WT, *OE11* and *OE15* Arabidopsis seedlings were harvested from the medium and subjected to 15% PEG treatment for 6 h and 12 h, respectively. After osmotic treatments, seedlings were fully immersed in 1 mg/mL DAB staining solution and incubated for 8 h at room temperature in darkness. After staining incubation, chlorophyll decolorization was performed using 95% (*v*/*v*) ethanol. Following full bleaching, samples were visualized and photographed under a light microscope.

For quantitative measurement of ROS levels by chemiluminescence, eight individual 7-day-old Arabidopsis seedlings cultivated on half-strength MS (1/2 MS) medium were transferred to a 96-well microplate and pre-incubated in 100 μL double-distilled water (ddH_2_O) for 8 h. A working solution containing 30% PEG, luminol and horseradish peroxidase (HRP) was freshly prepared, and 100 μL of this solution was applied to each well to impose a 6 h stress treatment. Chemiluminescence intensity was recorded with a microplate reader, and cumulative ROS production throughout the 6 h treatment was quantified.

### 4.7. Promoter Activity Analysis

A 2000 bp genomic sequence upstream of the *CiXTH18* start codon was amplified from *C. illinoinensis* genomic DNA and independently cloned into the *pCAMBIA1381* (*ProCiXTH18::GUS*) and the *pGreenII0800* vector (*ProCiXTH18::LUC*). Both constructs were introduced into *A. tumefaciens* GV3101 and infiltrated into N. benthamiana leaves. At 48 h post-infiltration, leaves were treated with 100 µM ABA dissolved in 0.1% (*v*/*v*) ethanol or an equivalent volume of solvent as control. Leaf disks were collected from three leaves per plant, across three independent plants, at 48 h post-treatment. GUS staining was performed by incubating disks in X-Gluc substrate solution overnight at 37 °C, followed by destaining in 70% ethanol. Luciferase activity was quantified using a luminescence imaging system.

### 4.8. Measurement of Water Loss Rate

For detached-leaf water-loss assays, fully expanded rosette leaves with similar developmental status were collected from 4-week-old WT, *OE11*, and *OE15* plants. Eight leaves were analyzed for each genotype. The initial fresh weight was recorded immediately after detachment, and the leaves were subsequently maintained at room temperature under the same environmental conditions. Fresh weights were recorded again at 2, 4, and 8 h after detachment. The water-loss rate was calculated as follows: water-loss rate (%) = [(W_0_ − W_t_)/W_0_] × 100%, where W_0_ represents the initial fresh weight and W_t_ represents the fresh weight at the corresponding time point.

### 4.9. Statistical Analysis

All experiments were performed with no fewer than three biological replicates, the details of which are indicated in the corresponding figure legends. Statistical analyses were conducted via one-way ANOVA, and all graphs were generated using GraphPad Prism 9.5 software. Root length and fluorescence intensity were quantified using ImageJ software. All experimental data were recorded, organized, and analyzed with Microsoft Excel.

## 5. Conclusions

In this study, a total of 34 *CiXTH* genes were identified at the genome-wide scale in *C. illinoinensis* and divided into four clades. Transcriptomic profiling identified *CiXTH18* as a gene with sustained transcriptional activation under drought treatment. Heterologous expression assays in Arabidopsis demonstrated that *CiXTH18* correlates with enhanced performance under PEG-mediated osmotic stress, including promoted primary root elongation, reduced detached leaf water loss, and alleviated stress-induced H_2_O_2_ accumulation. The expression of the stress-related gene *DREB2A* and ABA biosynthesis gene *NCED3* were elevated in *CiXTH18*-expressing lines upon osmotic treatment. This work outlines a potential regulatory module connecting *CiXTH18* expression, ROS homeostasis and ABA signaling during osmotic stress response, and provides *CiXTH18* as a candidate gene for further investigation into stress adaptation in woody nut crops.

## Data Availability

The data in the article can be found in the Supplementary Materials.

## Supplementary Materials

The following supporting information can be downloaded at: https://www.mdpi.com/article/10.3390/plants15152319/s1. Figure S1: Relative expression level analysis of *CiXTH18*. The expression level of *CIXTH18* in the *OE4*, 8, 11, 15 and 20 lines, with dots representing three biological replicates and each dot being the average of technical replicates; Table S1: Rename and physicochemical properties of CiXTH members, primer sequences used in this study.

## References

[1] Normile D. (2008). Reinventing rice to feed the world. Science.

[2] Boyer J.S. (1982). Plant productivity and environment. Science.

[3] Scharte J., Schön H., Tjaden Z., Weis E., von Schaewen A. (2009). Isoenzyme replacement of glucose-6-phosphate dehydrogenase in the cytosol improves stress tolerance in plants. Proc. Natl. Acad. Sci. USA.

[4] Waadt R., Seller C.A., Hsu P.K., Takahashi Y., Munemasa S., Schroeder J.I. (2022). Plant hormone regulation of abiotic stress responses. Nat. Rev. Mol. Cell Biol..

[5] Sies H., Jones D.P. (2020). Reactive oxygen species (ROS) as pleiotropic physiological signalling agents. Nat. Rev. Mol. Cell Biol..

[6] Hu C.H., Wang P.Q., Zhang P.P., Nie X.-M., Li B.-B., Tai L., Liu W.-T., Li W.-Q., Chen K.-M. (2020). NADPH Oxidases: The Vital Performers and Center Hubs during Plant Growth and Signaling. Cells.

[7] Medeiros D.B., Barros J.A.S., Fernie A.R., Araújo W.L. (2020). Eating Away at ROS to Regulate Stomatal Opening. Trends Plant Sci..

[8] Peters R.L., Arend M., Zahnd C., Hoch G., Arndt S.K., Cernusak L.A., Poyatos R., Zhorzel T., Kahmen A. (2025). Uniform regulation of stomatal closure across temperate tree species to sustain nocturnal turgor and growth. Nat. Plants.

[9] Schroeder J.I., Kwak J.M., Allen G.J. (2001). Guard cell abscisic acid signalling and engineering drought hardiness in plants. Nature.

[10] Zheng X., Kang S., Jing Y., Ren Z., Li L., Zhou J.-M., Berkowitz G., Shi J., Fu A., Lan W. (2018). Danger-Associated Peptides Close Stomata by OST1-Independent Activation of Anion Channels in Guard Cells. Plant Cell.

[11] Kim T.H., Böhmer M., Hu H., Nishimura N., Schroeder J.I. (2010). Guard cell signal transduction network: Advances in understanding abscisic acid, CO_2_, and Ca^2+^ signaling. Annu. Rev. Plant Biol..

[12] Wang W., Chen Q., Xu S., Liu W., Zhu X., Song C. (2020). Trehalose-6-phosphate phosphatase E modulates ABA-controlled root growth and stomatal movement in Arabidopsis. J. Integr. Plant Biol..

[13] Zhao Y., Zhang Z., Gao J., Wang P., Hu T., Wang Z., Hou Y.-J., Wan Y., Liu W., Xie S. (2018). Arabidopsis Duodecuple Mutant of PYL ABA Receptors Reveals PYL Repression of ABA-Independent SnRK2 Activity. Cell Rep..

[14] Pri-Tal O., Sun Y., Dadras A., Fürst-Jansen J.M.R., Zimran G., Michaeli D., Wijerathna-Yapa A., Shpilman M., Merilo E., Yarmolinsky D. (2024). Constitutive activation of ABA receptors in Arabidopsis reveals unique regulatory circuitries. New Phytol..

[15] Sandhu N., Singh A., Dixit S., Cruz M.T.S., Maturan P.C., Jain R.K., Kumar A. (2014). Identification and mapping of stable QTL with main and epistasis effect on rice grain yield under upland drought stress. BMC Genet.

[16] Parmar N., Singh K.H., Sharma D., Singh L., Kumar P., Nanjundan J., Khan Y.J., Chauhan D.K., Thakur A.K. (2017). Genetic engineering strategies for biotic and abiotic stress tolerance and quality enhancement in horticultural crops: A comprehensive review. 3 Biotech.

[17] Gechev T. (2025). Genomics control of biostimulant-induced stress tolerance and crop yield enhancement. Plant J..

[18] Chang Y., Fang Y., Liu J., Ye T., Li X., Tu H., Ye Y., Wang Y., Xiong L. (2024). Stress-induced nuclear translocation of ONAC023 improves drought and heat tolerance through multiple processes in rice. Nat. Commun..

[19] Xu C., Shan J., Liu T., Wang Q., Ji Y., Zhang Y., Wang M., Xia N., Zhao L. (2023). CONSTANS-LIKE 1a positively regulates salt and drought tolerance in soybean. Plant Physiol..

[20] Eklöf J.M., Brumer H. (2010). The XTH gene family: An update on enzyme structure, function, and phylogeny in xyloglucan remodeling. Plant Physiol..

[21] Campbell P., Braam J. (1999). Xyloglucan endotransglycosylases: Diversity of genes, enzymes and potential wall-modifying functions. Trends Plant Sci..

[22] Malko M.M., Yanfeng Z., Jiakun G., Yi W., Khanzada A., Xiao W., Li Q., Samo A., Cai J., Dong J. (2025). Dynamic hormone flux and gene-metabolite integration influence drought tolerance and tiller plasticity under drought conditions in wheat. Plant Physiol. Biochem..

[23] Richtmann L., Prochetto S., Thiébaut N., Sarthou M.C.M., Boutet S., Hanikenne M., Clemens S., Verbruggen N. (2025). Arabidopsis thaliana root responses to Cd exposure: Insights into root tip-specific changes and the role of HY5 in limiting Cd accumulation and promoting tolerance. Plant J..

[24] De Caroli M., Manno E., Piro G., Lenucci M.S. (2021). Ride to cell wall: Arabidopsis XTH11, XTH29 and XTH33 exhibit different secretion pathways and responses to heat and drought stress. Plant J..

[25] Tan Y., Zhan H., Chen H., Li X., Chen C., Liu H., Chen Y., Zhao Z., Xiao Y., Liu J. (2024). Genome-wide identification of XTH gene family in Musa acuminata and response analyses of MaXTHs and xyloglucan to low temperature. Physiol. Plant..

[26] Yokoyama R., Nishitani K. (2001). A comprehensive expression analysis of all members of a gene family encoding cell-wall enzymes allowed us to predict cis-regulatory regions involved in cell-wall construction in specific organs of Arabidopsis. Plant Cell Physiol..

[27] Yokoyama R., Rose J.K., Nishitani K. (2004). A surprising diversity and abundance of xyloglucan endotransglucosylase/hydrolases in rice. Classification and expression analysis. Plant Physiol..

[28] Geisler-Lee J., Geisler M., Coutinho P.M., Segerman B., Nishikubo N., Takahashi J., Aspeborg H., Djerbi S., Master E., Andersson-Gunnerås S. (2006). Poplar carbohydrate-active enzymes. Gene identification and expression analyses. Plant Physiol..

[29] Fu M.M., Liu C., Wu F. (2019). Genome-Wide Identification, Characterization and Expression Analysis of Xyloglucan Endotransglucosylase/Hydrolase Genes Family in Barley (Hordeum vulgare). Molecules.

[30] Yang Z., Zhang R., Zhou Z. (2022). The XTH Gene Family in Schima superba: Genome-Wide Identification, Expression Profiles, and Functional Interaction Network Analysis. Front. Plant Sci..

[31] Yang B., Yang C., Chen J., Ren H., Wang K., Liu L., Yao X. (2025). CiNAC2 positively regulates drought stress tolerance by promoting superoxide dismutase activity in pecan (*Carya illinoinensis*). Hortic. Plant J..

[32] Marco R., Goldschmidt R.J.Z., Herter F.G., Martins C.R., Mello-Farias P.C., Uberti A. (2021). The irrigation effect on nuts’ growth and yield of *Carya illinoinensis*. An. Acad. Bras. Cienc..

[33] Chaves M.M., Maroco J.P., Pereira J.S. (2003). Understanding plant responses to drought—From genes to the whole plant. Funct. Plant Biol..

[34] Behar H., Graham S.W., Brumer H. (2018). Comprehensive cross-genome survey and phylogeny of glycoside hydrolase family 16 members reveals the evolutionary origin of EG16 and XTH proteins in plant lineages. Plant J..

[35] Baumann M.J., Eklof J.M., Michel G., Kallas A.M., Teeri T.T., Czjzek M., Brumer H. (2007). Structural evidence for the evolution of xyloglucanase activity from xyloglucan endo-transglycosylases: Biological implications for cell wall metabolism. Plant Cell.

[36] Zhu K., Fan P., Liu H., Tan P., Ma W., Mo Z., Zhao J., Chu G., Peng F. (2022). Insight into the CBL and CIPK gene families in pecan (*Carya illinoinensis*): Identification, evolution and expression patterns in drought response. BMC Plant Biol..

[37] Cosgrove D.J. (2005). Growth of the plant cell wall. Nat. Rev. Mol. Cell Biol..

[38] Rose J.K., Braam J., Fry S.C., Nishitani K. (2002). The XTH family of enzymes involved in xyloglucan endotransglucosylation and endohydrolysis: Current perspectives and a new unifying nomenclature. Plant Cell Physiol..

[39] Park Y.B., Cosgrove D.J. (2015). Xyloglucan and its interactions with other components of the growing cell wall. Plant Cell Physiol..

[40] Bi H., Liu Z., Liu S., Qiao W., Zhang K., Zhao M., Wang D. (2024). Genome-wide analysis of wheat xyloglucan endotransglucosylase/hydrolase (XTH) gene family revealed TaXTH17 involved in abiotic stress responses. BMC Plant Biol..

[41] Yuan W., Yao F., Liu Y., Xiao H., Sun S., Jiang C., An Y., Chen N., Huang L., Lu M. (2024). Identification of the xyloglucan endotransglycosylase/hydrolase genes and the role of PagXTH12 in drought resistance in poplar. For. Res..

[42] Ma S., Li T., Feng Z., Zhang Y., Jiang H., Li Y. (2026). MdXTH30, an apple gene encoding endotransferase/hydrolase for xyloglucan, enhances plant resistance to drought, salt and pathogenic stresses. J. Integr. Agric..

[43] Uga Y., Sugimoto K., Ogawa S., Rane J., Ishitani M., Hara N., Kitomi Y., Inukai Y., Ono K., Kanno N. (2013). Control of root system architecture by DEEPER ROOTING 1 increases rice yield under drought conditions. Nat. Genet..

[44] Cosgrove D.J. (2016). Plant cell wall extensibility: Connecting plant cell growth with cell wall structure, mechanics, and the action of wall-modifying enzymes. J. Exp. Bot..

[45] Fry S.C. (2004). Primary cell wall metabolism: Tracking the careers of wall polymers in living plant cells. New Phytol..

[46] Qi J., Song C.P., Wang B., Zhou J., Kangasjärvi J., Zhu J., Gong Z. (2018). Reactive oxygen species signaling and stomatal movement in plant responses to drought stress and pathogen attack. J. Integr. Plant Biol..

[47] Waszczak C., Carmody M., Kangasjärvi J. (2018). Reactive Oxygen Species in Plant Signaling. Annu. Rev. Plant Biol..

[48] Yang Y., Xu L., Hao C., Wan M., Tao Y., Zhuang Y., Su Y., Li L. (2024). The microRNA408-plantacyanin module balances plant growth and drought resistance by regulating reactive oxygen species homeostasis in guard cells. Plant Cell.

[49] Wang J., Nan N., Shi L., Li N., Huang S., Zhang A., Liu Y., Guo P., Liu B., Xu Z. (2020). Arabidopsis BRCA1 represses RRTF1-mediated ROS production and ROS-responsive gene expression under dehydration stress. New Phytol..

[50] Zhang X., Zhang D., Zhong C., Li W., Dinesh-Kumar S.P., Zhang Y. (2025). Orchestrating ROS regulation: Coordinated post-translational modification switches in NADPH oxidases. New Phytol..

[51] Gayen D., Kumar S., Barua P., Lande N.V., Karmakar S., Dey A.K., Gayali S., Maiti T.K., Molla K.A., Murumkar S. (2025). OsDUF2488 acts synergistically with OsPrx1.1, regulates ROS metabolism and promotes dehydration tolerance in rice. Plant Biotechnol. J..

[52] Hsu P.K., Dubeaux G., Takahashi Y., Schroeder J.I. (2021). Signaling mechanisms in abscisic acid-mediated stomatal closure. Plant J..

[53] Kwak J.M., Mori I.C., Pei Z.M., Leonhardt N., Torres M.A., Dangl J.L., Bloom R.E., Bodde S., Jones J.D., Schroeder J.I. (2003). NADPH oxidase AtrbohD and AtrbohF genes function in ROS-dependent ABA signaling in Arabidopsis. Embo J..

[54] Pei Z.M., Murata Y., Benning G., Thomine S., Klüsener B., Allen G.J., Grill E., Schroeder J.I. (2000). Calcium channels activated by hydrogen peroxide mediate abscisic acid signalling in guard cells. Nature.

[55] Sakuma Y., Maruyama K., Qin F., Osakabe Y., Shinozaki K., Yamaguchi-Shinozaki K. (2006). Dual function of an Arabidopsis transcription factor DREB2A in water-stress-responsive and heat-stress-responsive gene expression. Proc. Natl. Acad. Sci. USA.

[56] Qin F., Sakuma Y., Tran L.S., Maruyama K., Kidokoro S., Fujita Y., Fujita M., Umezawa T., Sawano Y., Miyazono K.-I. (2008). Arabidopsis DREB2A-interacting proteins function as RING E3 ligases and negatively regulate plant drought stress-responsive gene expression. Plant Cell.

[57] Sato H., Takasaki H., Takahashi F., Maruyama K., Kidokoro S., Fujita Y., Fujita M., Umezawa T., Sawano Y., Miyazono K.-I. (2018). Arabidopsis thaliana NGATHA1 transcription factor induces ABA biosynthesis by activating NCED3 gene during dehydration stress. Proc. Natl. Acad. Sci. USA.

[58] Hao G.P., Zhang X.H., Wang Y.Q., Wu Z., Huang C. (2009). Nucleotide variation in the NCED3 region of Arabidopsis thaliana and its association study with abscisic acid content under drought stress. J. Integr. Plant Biol..

[59] Tenhaken R. (2014). Cell wall remodeling under abiotic stress. Front. Plant Sci..

[60] Esmaeilzadeh-Moridani M., Esfahani M., Aalami A., Moumeni A., Khaledian M., Chaleshtori M.H. (2023). Expression profiling of yield related genes in rice cultivars under terminal drought stress. Mol. Biol. Rep..

[61] Murashige T., Skoog F. (1962). A Revised Medium for Rapid Growth and Bio Assays with Tobacco Tissue Cultures. Physiol. Plant..

[62] Potter S.C., Luciani A., Eddy S.R., Park Y., López R., Finn R.D. (2018). HMMER web server: 2018 update. Nucleic Acids Res..

[63] Letunic I., Khedkar S., Bork P. (2021). SMART: Recent updates, new developments and status in 2020. Nucleic Acids Res..

[64] Wilkins M.R., Gasteiger E., Bairoch A., Hoogland C., Gattiker A., Duvaud S.E., Appel R.D. (1999). Protein identification and analysis tools in the ExPASy server. Methods Mol. Biol..

[65] Tamura K., Stecher G., Kumar S. (2021). MEGA11: Molecular Evolutionary Genetics Analysis Version 11. Mol. Biol. Evol..

[66] Li Y., Yang Y., Hu Y., Liu H., He M., Yang Z., Kong F., Liu X., Hou X. (2019). DELLA and EDS1 Form a Feedback Regulatory Module to Fine-Tune Plant Growth-Defense Tradeoff in Arabidopsis. Mol. Plant.

